# Supplementary material for: An artificial intelligence system for predicting the deterioration of COVID-19 patients in the emergency department
Source: NPJ Digit Med. 2021 May 12;4:80. doi: 10.1038/s41746-021-00453-0 (PMC8115328; doi:10.1038/s41746-021-00453-0)
Supplement: Supplementary file 1 — Reporting Summary [file 41746_2021_453_MOESM1_ESM.pdf]

nature research

Corresponding author(s): Krzysztof Geras
Last updated by author(s): Jan 31, 2021

Reporting Summary

Nature Research wishes to improve the reproducibility of the work that we publish. This form provides structure for consistency and transparency in reporting. For further information on Nature Research policies, see our Editorial Policies and the Editorial Policy Checklist.

Statistics

For all statistical analyses, confirm that the following items are present in the figure legend, table legend, main text, or Methods section.

n/a	Confirmed	
☐	☒	The exact sample size (*n*) for each experimental group/condition, given as a discrete number and unit of measurement
☐	☒	A statement on whether measurements were taken from distinct samples or whether the same sample was measured repeatedly
☐	☒	The statistical test(s) used AND whether they are one- or two-sided<br>*Only common tests should be described solely by name; describe more complex techniques in the Methods section.*
☐	☒	A description of all covariates tested
☒	☐	A description of any assumptions or corrections, such as tests of normality and adjustment for multiple comparisons
☐	☒	A full description of the statistical parameters including central tendency (e.g. means) or other basic estimates (e.g. regression coefficient) AND variation (e.g. standard deviation) or associated estimates of uncertainty (e.g. confidence intervals)
☐	☒	For null hypothesis testing, the test statistic (e.g. *F*, *t*, *r*) with confidence intervals, effect sizes, degrees of freedom and *P* value noted<br>*Give P values as exact values whenever suitable.*
☒	☐	For Bayesian analysis, information on the choice of priors and Markov chain Monte Carlo settings
☒	☐	For hierarchical and complex designs, identification of the appropriate level for tests and full reporting of outcomes
☒	☐	Estimates of effect sizes (e.g. Cohen's *d*, Pearson's *r*), indicating how they were calculated

Our web collection on statistics for biologists contains articles on many of the points above.

Software and code

Policy information about availability of computer code

Data collection	Data collection was performed by independent members of NYU Langone Health. The images were provided in DICOM format while the clinical data was provided in CSV files.
Data analysis	Python (3.6), PyTorch (1.1.0), torchvision (0.2.2), NumPy (1.14.3), SciPy (1.0.0), H5py (2.7.1), imageio (2.4.1), pandas (0.22.0), opencv-python (3.4.2), tqdm (4.19.8), matplotlib (3.0.2), LightGBM (2.3.1). The custom code is provided in the shared repository. Details of the methodology can be found in the Methods section.

For manuscripts utilizing custom algorithms or software that are central to the research but not yet described in published literature, software must be made available to editors and reviewers. We strongly encourage code deposition in a community repository (e.g. GitHub). See the Nature Research guidelines for submitting code & software for further information.

Data

Policy information about availability of data

All manuscripts must include a data availability statement. This statement should provide the following information, where applicable:

- Accession codes, unique identifiers, or web links for publicly available datasets
- A list of figures that have associated raw data
- A description of any restrictions on data availability

The datasets analysed during the current study were collected and stored at NYU Langone Health. The data is not publicly available and restrictions apply to its use based on local ethical approvals.

Field-specific reporting

Please select the one below that is the best fit for your research. If you are not sure, read the appropriate sections before making your selection.

☒ Life sciences ☐ Behavioural & social sciences ☐ Ecological, evolutionary & environmental sciences

For a reference copy of the document with all sections, see nature.com/documents/nr-reporting-summary-flat.pdf

Life sciences study design

All studies must disclose on these points even when the disclosure is negative.

Sample size	The final training and test datasets consisted of data collected from 3,661 patients, who tested positive for COVID-19 at NYU Langone Health, recorded between March 3 and May 15.
Data exclusions	We excluded chest X-ray images that had missing radiology reports or patient encounter information to ensure data completeness, as well as chest X-ray images that were collected after a patient had experienced an adverse event, since deterioration had already occurred.<br>We included patients who were discharged, and patients who had experienced in-hospital mortality, in order to obtain a full record of adverse events. We also manually checked for images of already intubated patients, and excluded them. In the test set, we only included images collected in the ED, and excluded images collected during inpatient encounters.
Replication	All models were trained for several runs to ensure reproducibility and we reported the results of the ensemble of all models.
Randomization	The data was randomly split into a training set (%) and a test set (%). The data from a single patient could only appear in the training set or test set.
Blinding	This is not relevant to our study because the research team was not involved with the data collection process.

Reporting for specific materials, systems and methods

We require information from authors about some types of materials, experimental systems and methods used in many studies. Here, indicate whether each material, system or method listed is relevant to your study. If you are not sure if a list item applies to your research, read the appropriate section before selecting a response.

Materials & experimental systems

n/a	Involved in the study	
☒	☐	Antibodies
☒	☐	Eukaryotic cell lines
☒	☐	Palaeontology and archaeology
☒	☐	Animals and other organisms
☐	☒	Human research participants
☒	☐	Clinical data
☒	☐	Dual use research of concern

Methods

n/a	Involved in the study	
☒	☐	ChIP-seq
☒	☐	Flow cytometry
☒	☐	MRI-based neuroimaging

Human research participants

Policy information about studies involving human research participants

Population characteristics	The data was collected in New York, USA. The training set consisted of 5,617 chest X-ray images collected from 2,943 unique patients, where 41.0% of patients were female with mean age (standard deviation) of 62.9 (17.2). The test set consisted of 832 chest X-ray images collected from 718 unique patients, where 42.5% of patients were female with mean age (standard deviation) of 64.9 (17.2).
Recruitment	This was a retrospective study and therefore no recruitment process was involved.
Ethics oversight	This study was approved by the Institutional Review Board at NYU Langone Health

Note that full information on the approval of the study protocol must also be provided in the manuscript.
