# Supplementary material for: An artificial intelligence system for predicting the deterioration of COVID-19 patients in the emergency department
Source: NPJ Digit Med. 2021 May 12;4:80. doi: 10.1038/s41746-021-00453-0 (PMC8115328; doi:10.1038/s41746-021-00453-0)
Supplement: Supplementary file 2 — Supplementary Information [file 41746_2021_453_MOESM2_ESM.pdf]

Supplementary Information

Supplementary Note 1: Additional results

We visualize the receiver operating characteristic (ROC) and precision-recall (PR) curves on the test set in Supplementary Figure 1. In **a**, we group the results based on the predictive models (COVID-GMIC, COVID-GBM, and the ensemble of both), while in **b**, we group the performances based on the time window of the task (i.e., 24, 48, 72, and 96 hours). In Supplementary Figure 5, we visualize the ROC and PR curves on the test set considered in the reader study.

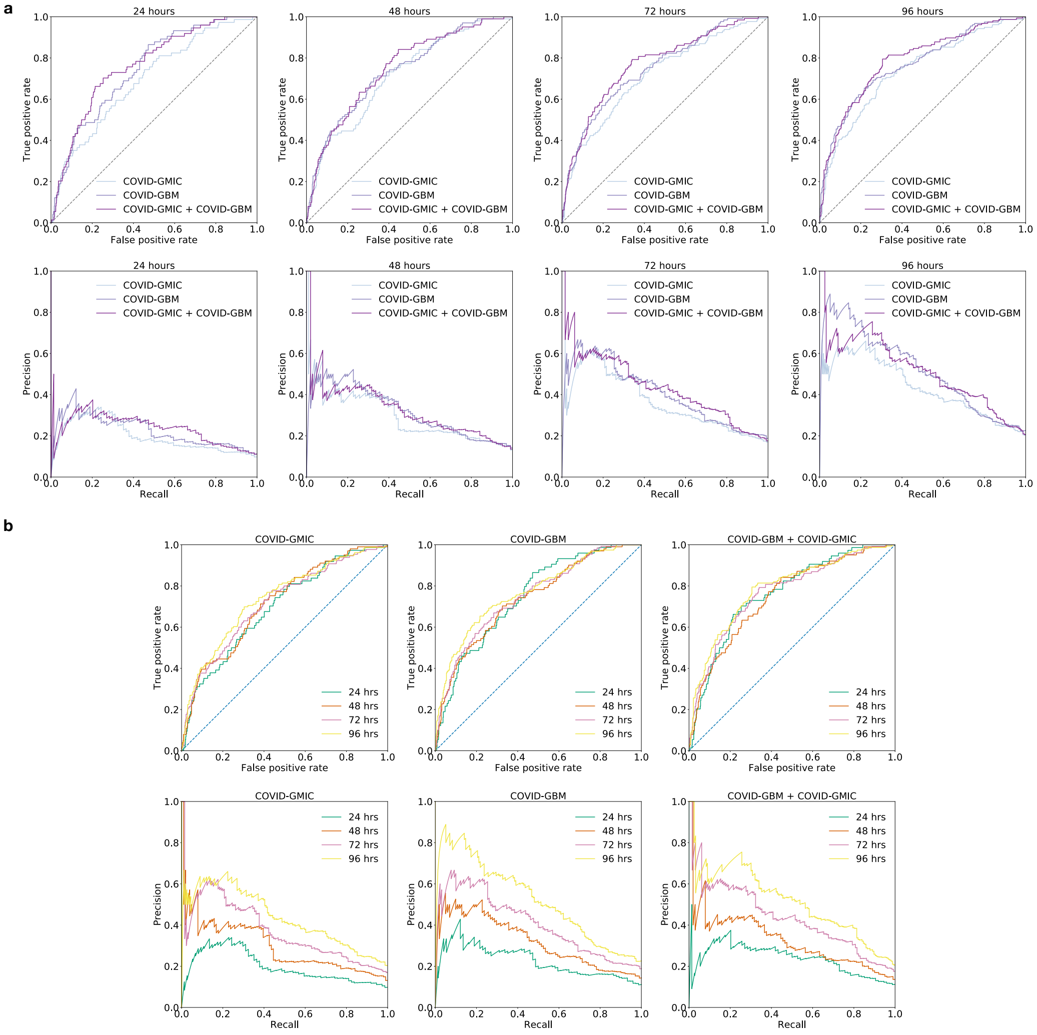

Supplementary Figure 1: **Receiver operating characteristic (ROC) and Precision-Recall (PR) curves for predicting the onset of adverse events within 24, 48, 72, and 96 hours evaluated on the test set. a**, ROC and PR curves are grouped by predictive models. Ensembling COVID-GMIC and COVID-GBM improves performance in almost all cases. **b**, ROC and PR curves are grouped by time window of the task. The AUC and PR AUC improve as the length of the time window increases, which is consistence across models. Numerical values of AUCs and PR AUCs can be found in Table 2.

Supplementary Table 1: Positive predictive values (PPV) and negative predictive values (NPV) of the outcome classification task on the held-out test set (n represents the number of images). We include 95% confidence intervals estimated by 1,000 iterations of the bootstrap method [30]. COVID-GBM achieves the best performance across all time windows in terms of the PPV and NPV.

	Test set (n=832)							
	NPV				PPV			
	24 hours	48 hours	72 hours	96 hours	24 hours	48 hours	72 hours	96 hours
COVID-GBM	100% (100%, 100%)	98.9% (96.6%, 100%)	99.1% (96.5%, 100%)	96.2% (93.0%, 99.2%)	10.4% (8.3%, 12.6%)	14.8% (12.4%, 17.6%)	19.7% (16.8%, 22.8%)	23.8% (20.4%, 27.2%)
COVID-GMIC	98.3% (94.6%, 100%)	98.9% (95.6%, 100%)	93.8% (89.7%, 97.5%)	93.8% (89.1%, 96.8%)	10.3% (8.2%, 12.8%)	14.8% (12.1%, 17.9%)	18.8% (15.9%, 22.6%)	23.3% (20.1%, 28%)
COVID-GBM + COVID-GMIC	100% (100%, 100%)	98.9% (95.5%, 100%)	95.6% (92.0%, 99.1%)	96.2% (92.2%, 99.2%)	10.4% (8.2%, 12.8%)	14.8% (12.0%, 17.6%)	19.1% (16.1%, 22.2%)	23.8% (20.5%, 27.1%)

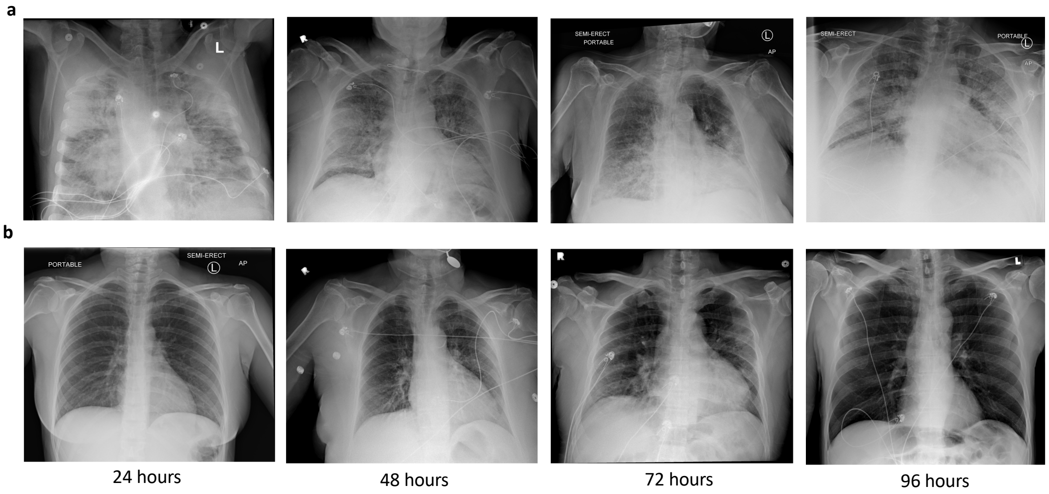

Supplementary Figure 2: **Examples that were incorrectly classified by COVID-GMIC for predicting the risk of deterioration over 24, 48, 72, and 96 hours (columns). a,** False positive examples. Based on clinical assessment, the chest X-rays are abnormal and therefore it is not unreasonable that the model predicted a higher likelihood of deterioration. It is likely that the patients would have had a guarded prognosis with this chest X-ray severity and were therefore perhaps prioritized in terms of care. Additionally, our deterioration window only went up to 96 hours and those may patients may have deteriorated beyond the 96 hour window. **b,** False negative examples. Most of the chest X-rays show no, or at most moderate, airspace opacities. It is likely that those patients may have had non-respiratory deterioration, such as cardiovascular or neurologic complications that led to further deterioration.

The average importance of the top ten features computed by the COVID-GBM models are shown in Supplementary Figure 3.a. The importance of a feature is measured by the numbers of times the feature is used to split the data across all trees in a single COVID-GBM model. Age is amongst the top ten features across all time windows, which is consistent with existing findings that mortality is more common amongst elderly COVID-19 patients than younger patients [64]. The inclusion of the vital sign variables, amongst the top ten features across all models, is also aligned with existing research suggesting that they are strong indicators of deterioration [20].

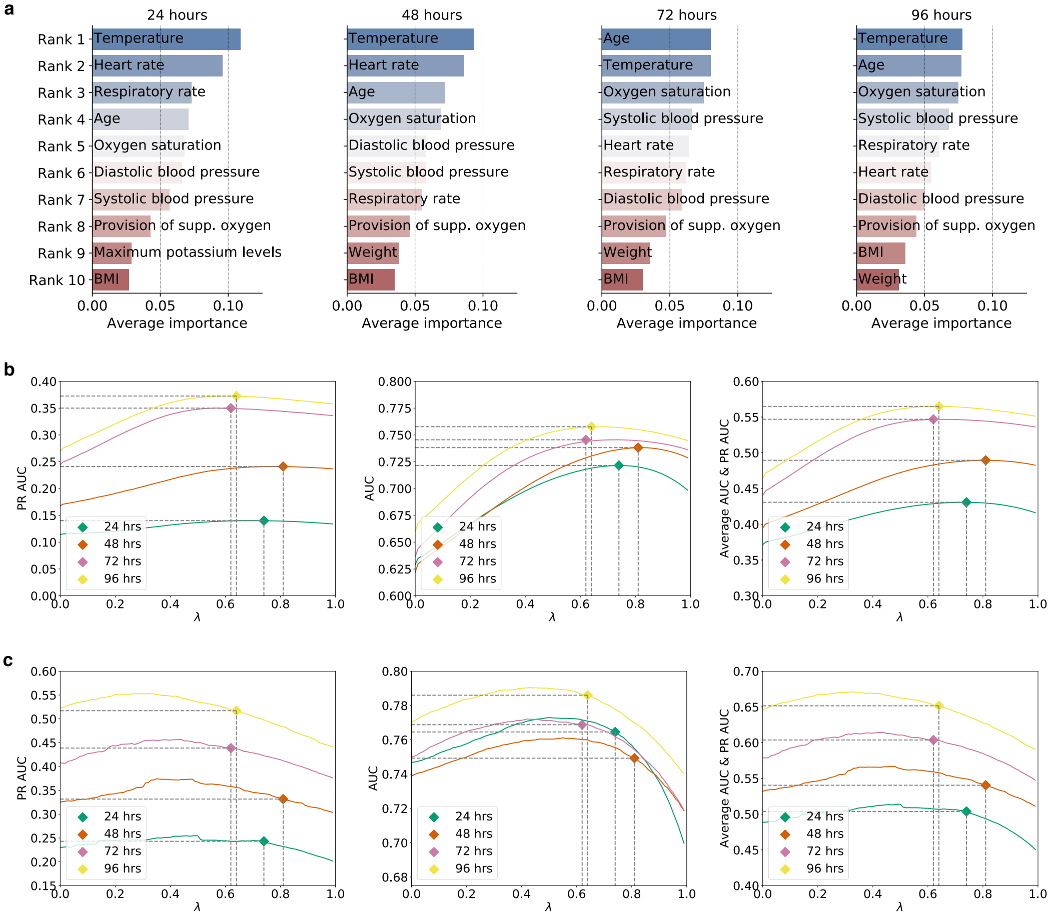

Supplementary Figure 3: **Additional results for COVID-GBM and the ensemble of COVID-GBM and COVID-GMIC. a**, The average importance of the top ten features computed by the nine COVID-GBM ensemble models for 24, 48, 72, and 96 hours. The importance of a feature is measured by the numbers of times the feature is used to split the data across all trees in a model. **b**, The effect of varying λ, the weight on the COVID-GMIC prediction, in combining the predictions of COVID-GMIC and COVID-GBM when using AUC, PR AUC and the average AUC and PR AUC on the validation set. For the average AUC and PR AUC, the optimal λ was 0.74 for 24 hours, 0.81 for 48 hours, 0.62 for 72 hours, and 0.64 for 96 hours. **c**, the optimal values of λ selected through the validation set in **b** are shown for the test set.

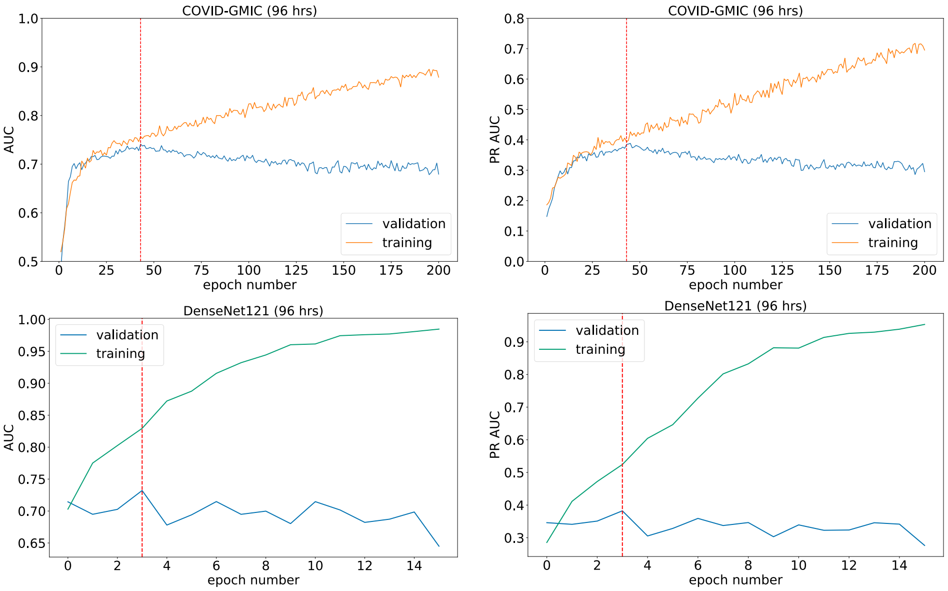

Supplementary Figure 4: **AUC and PR AUC for predicting clinical deterioration within 96 hours during training achieved by a selected COVID-GMIC model and a selected DenseNet121 model on the training and validation set.** We select the best epoch (marked in red) in which the model achieves highest AUC on the validation set. Our model selection mechanism ensures that the selected model is sufficiently trained and does not lie in the overfitted regime. We observe similar trends in the learning curves for predicting clinical deterioration within 24, 48, and 72 hours.

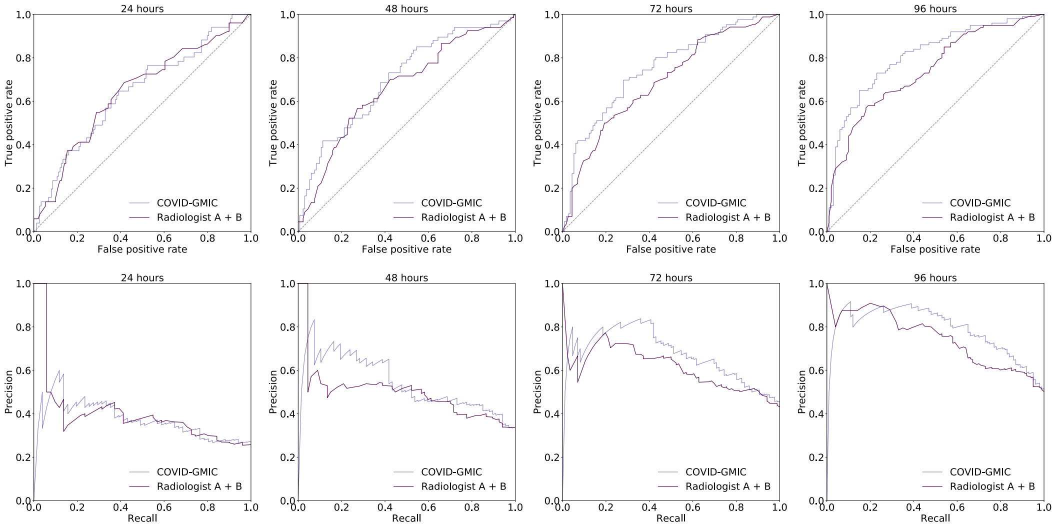

Supplementary Figure 5: **Test set ROC (top) and PR (bottom) curves of COVID-GMIC and the radiologists for predicting the risk of deterioration over 24, 48, 72, and 96 hours.** These results suggest that COVID-GMIC performs comparably to the radiologists. Numerical values of AUCs and PR AUCs can be found in Table 2.

In Supplementary Table 2, we show the concordance index results across all time intervals for the best DenseNet-121 and COVID-GMIC-DRC models.

Supplementary Table 2: Concordance index (with 95% confidence intervals) of the DRC curves generated by the best DenseNet-121 and COVID-GMIC-DRC models. Both models use input images of size 512×512 and are pretrained on the ChestX-ray14 dataset [59]. The results shows that the concordance index does not change much with the choice of time reference.

	Concordance index								
Time (in hours)	3	12	24	48	72	96	144	192	Ave.
DenseNet-121	0.681<br>(0.647, 0.714)	0.694<br>(0.658, 0.727)	0.701<br>(0.666, 0.735)	0.702<br>(0.667, 0.735)	0.703<br>(0.668, 0.734)	0.705<br>(0.671, 0.737)	0.706<br>(0.672, 0.739)	0.705<br>(0.67, 0.737)	0.701<br>(0.667, 0.733)
COVID-GMIC-DRC	0.692<br>(0.661, 0.734)	0.698<br>(0.664, 0.736)	0.706<br>(0.672, 0.74)	0.710<br>(0.677, 0.746)	0.712<br>(0.676, 0.745)	0.713<br>(0.678, 0.747)	0.716<br>(0.681, 0.748)	0.715<br>(0.68, 0.748)	**0.708**<br>(0.674, 0.741)

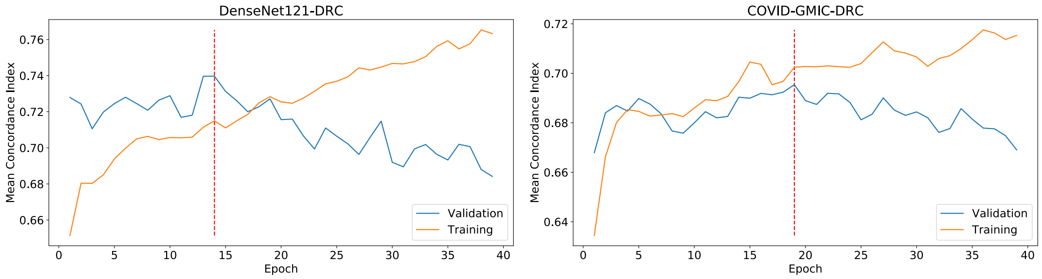

Supplementary Figure 6: **Training curves showing mean concordance index for DenseNet121 and COVID-GMIC-DRC models.** We select the best epoch (marked in red) in which the model achieves highest mean concordance index on the validation set. Our model selection mechanism ensures that the selected model is sufficiently trained and does not lie in the overfitted regime.

Supplementary Note 2: Image preprocessing

In Supplementary Figure 7.a, we show the heights and widths of the images prior to data augmentation. In Supplementary Figure 7.b, we show an example of a raw image and the final image after applying the preprocessing steps in Supplementary Figure 7.c.

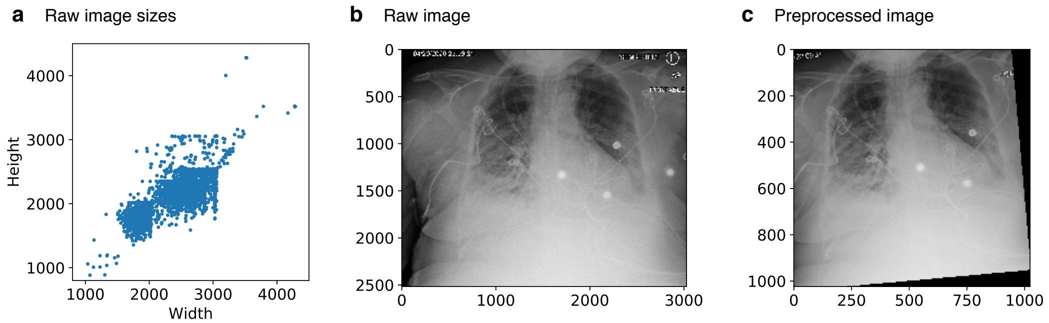

Supplementary Figure 7: **Additional information regarding the chest X-ray images. a,** Heights and widths (in pixels) of images prior to data augmentation. **b,** An example raw image. **c,** To ensure that the inputs to the model have a consistent size, we perform center cropping and rescaling. In addition, we apply random horizontal flipping, rotation, and translation to augment the training dataset.

Supplementary Note 3: Ablation studies

DenseNet-121-based models. DenseNet [48] is a deep neural network architecture which consists of dense blocks in which layers are directly connected to every other layer in a feed-forward fashion. It achieves strong performance on benchmark natural images dataset, such as CIFAR10/100 [65] and ILSVRC 2012 (ImageNet) dataset [66] while being computationally efficient. Here we compare COVID-GMIC to a specific variant of DenseNet, DenseNet-121, which has been applied to process chest X-ray images in the literature [49, 50, 51, 52].

The model assumes an input size of 224×224. We applied DenseNet-121-based models to predict deterioration and also to compute deterioration risk curves. We initialized the models with weights pretrained on the ChestX-ray14 dataset [59], provided at https://github.com/arnoweng/CheXNet. We used weight decay in the optimizer. To perform hyperparameter search, we sampled the learning rate and the rate of weight decay per step uniformly on a logarithmic scale between $10^{[-6,-1]}$ and $10^{[-6,-3]}$.

For adverse event prediction, the DenseNet-121 based model yielded test AUCs of 0.687 (95% CI: 0.621 - 0.749), 0.709 (95% CI: 0.653 - 0.757), 0.710 (95% CI: 0.660 - 0.763), and 0.736 (95% CI: 0.691 - 0.782), and PRAUCs of 0.216 (95% CI: 0.155 - 0.317), 0.315 (95% CI: 0.239 - 0.419), 0.373 (95% CI: 0.300 - 0464), and 0.454 (95% CI: 0.384 - 0.542) for 24, 48, 72, and 96 hours. The deterioration risk curves produced by the DenseNet-121 based models and the corresponding reliability plot are presented in Supplementary Figure 8.

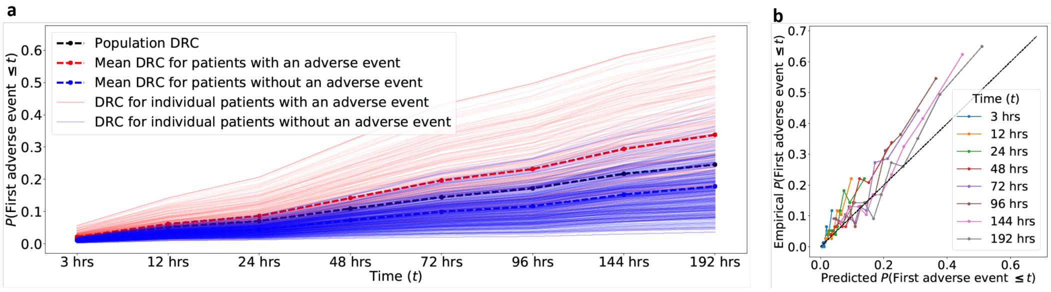

Supplementary Figure 8: **Deterioration risk curves (DRCs) and reliability plot for DenseNet-121.** This can be compared to Figure 4 in the main manuscript, which shows analogous graphs for COVID-GMIC-DRC. **a**, DRCs generated by DenseNet-121 model for patients in the test set with (faded red lines) and without adverse events (faded blue lines). The mean DRC for patients with adverse events (red dashed line) is higher than the DRC for patients without adverse events (blue dashed line) at all times. The graph also includes the ground-truth population DRC (black dashed line) computed from the test data. **b**, Reliability plot of the DRCs generated by DenseNet-121 model for patients in the test set. The empirical probabilities are computed by dividing the patients into deciles according to the value of the DRC at each time t. The empirical probability equals the fraction of patients in each decile that suffered adverse events up to t. This is plotted against the predicted probability, which equals the mean DRC of the patients in the decile. The diagram shows that these values are similar across the different values of t, and hence the model is well-calibrated (for comparison, perfect calibration would correspond to the diagonal black dashed line).

Impact of training set size. We evaluated the impact of the sample size used for training our machine learning models. Specifically, we evaluated our models on a subset of the training data, obtained by randomly sampling 12.5%, 25%, and 50% of the exams. Table 3 presents the AUCs and PR AUCs and the concordance indices achieved on the test set. It is evident that the performance of COVID-GMIC and COVID-GBM improve when increasing the number of images and clinical variables used for training, which highlights the importance of using a large dataset.

Supplementary Table 3: Model performance with 95% confidence intervals when using 12.5%, 25%, 50%, and 100% of the training data. We report AUCs for each time window in the adverse event prediction task. When evaluating the deterioration risk curves, we report the concordance index with a reference time of 96 hours, as well as the average of the index over all discretized times (3, 12, 24, 48, 72, 96, 144, and 192 hours).

		AUC / PR AUC				Concordance index	
		24 hours	48 hours	72 hours	96 hours	96 hours	Average
DenseNet-121	12.5%	0.608 (0.530, 0.678) / 0.182 (0.094, 0.241)	0.653 (0.594, 0.711) / 0.265 (0.177, 0.332)	0.672 (0.617, 0.722) / 0.336 (0.248, 0.401)	0.703 (0.654, 0.749) / 0.415 (0.330, 0.486)	0.675 (0.640, 0.708)	0.670 (0.636, 0.703)
	25%	0.638 (0.570, 0.708) / 0.174 (0.090, 0.227)	0.678 (0.621, 0.737) / 0.266 (0.170, 0.327)	0.682 (0.628, 0.734) / 0.327 (0.239, 0.393)	0.711 (0.662, 0.758) / 0.408 (0.321, 0.475)	0.676 (0.641, 0.709)	0.671 (0.637, 0.704)
	50%	0.672 (0.605, 0.737) / 0.214 (0.109, 0.278)	0.699 (0.644, 0.752) / 0.303 (0.209, 0.373)	0.698 (0.646, 0.747) / 0.351 (0.265, 0.417)	0.725 (0.679, 0.769) / 0.433 (0.349, 0.501)	0.694 (0.660, 0.728)	0.691 (0.657, 0.725)
	100%	**0.687** (0.621, 0.753) / **0.216** (0.154, 0.317)	**0.709** (0.654, 0.763) / **0.315** (0.239, 0.417)	**0.710** (0.658, 0.761) / **0.373** (0.298, 0.475)	**0.736** (0.689, 0.781) / **0.454** (0.377, 0.552)	**0.705** (0.673,0.739)	**0.701** (0.669,0.735)
COVID-GMIC	12.5%	0.640 (0.577, 0.703) / 0.145 (0.084, 0.180)	0.672 (0.621, 0.726) / 0.231 (0.146, 0.283)	0.677 (0.631, 0.728) / 0.318 (0.230, 0.387)	0.695 (0.652, 0.738) / 0.384 (0.294, 0.449)	0.673 (0.640, 0.706)	0.668 (0.635, 0.701)
	25%	0.661 (0.598, 0.724) / 0.177 (0.091, 0.229)	0.672 (0.616, 0.726) / 0.254 (0.162, 0.312)	0.677 (0.627, 0.723) / 0.327 (0.238, 0.388)	0.693 (0.649, 0.738) / 0.395 (0.313, 0.461)	0.689 (0.655, 0.723)	0.680 (0.646, 0.714)
	50%	0.646 (0.576, 0.715) / 0.164 (0.090, 0.212)	0.681 (0.624, 0.740) / 0.266 (0.172, 0.333)	0.687 (0.635, 0.742) / 0.351 (0.257, 0.428)	0.716 (0.669, 0.764) / 0.424 (0.332, 0.502)	0.699 (0.664, 0.733)	0.690 (0.657, 0.722)
	100%	**0.695** (0.626, 0.753) / **0.200** (0.142, 0.276)	**0.716** (0.663, 0.769) / **0.302** (0.230, 0.395)	**0.717** (0.667, 0.767) / **0.374** (0.297, 0.461)	**0.738** (0.693, 0.782) / **0.439** (0.363, 0.521)	**0.713** (0.679,0.748)	**0.708** (0.675,0.742)
COVID-GBM	12.5%	0.674 (0.609, 0.736) / 0.262 (0.153, 0.344)	0.699 (0.647, 0.753) / 0.297 (0.199, 0.366)	0.710 (0.666, 0.761) / 0.395 (0.310, 0.472)	0.708 (0.663, 0.755) / 0.439 (0.361, 0.516)		
	25%	0.688 (0.628, 0.740) / 0.175 (0.102, 0.220)	0.716 (0.666, 0.765) / 0.319 (0.227, 0.401)	0.733 (0.689, 0.778) / 0.385 (0.304, 0.461)	0.739 (0.695, 0.784) / 0.476 (0.402, 0.545)		
	50%	0.743 (0.699, 0.796) / 0.210 (0.119, 0.263)	**0.752** (0.702, 0.797) / **0.325** (0.252, 0.425)	0.749 (0.706, 0.795) / 0.418 (0.326, 0.495)	0.751 (0.711, 0.796) / 0.482 (0.396, 0.557)		
	100%	**0.747** (0.692, 0.798) / **0.230** (0.167, 0.322)	0.739 (0.687, 0.793) / **0.325** (0.253, 0.425)	**0.750** (0.704, 0.794) / **0.408** (0.334, 0.502)	**0.770** (0.728, 0.811) / **0.523** (0.439, 0.611)		

Impact of input image resolution. Prior work on deep learning for medical images [67] report that using high resolution input images can improve performance. In this section, we analyze the impact of image resolution on our tasks of interest. We consider the following image sizes: 128×128, 256×256, 512×512, and 1024×1024. We pretrain all models on the ChestX-ray14 dataset [59] and then fine-tune them on our dataset. Results on the test set are reported in Supplementary Table 4.

The DenseNet-121 based model achieves the best AUCs when using an image size of 256×256, and the best concordance index for 512×512. Further increasing the resolution does not improve performance. COVID-GMIC achieves the best performance for the highest input image resolution of 1024×1024, while achieving the best concordance index for 512×512. While a further increase in performance may be possible, we did not consider any larger image sizes resolutions because the computational cost would become prohibitively high.

Supplementary Table 4: Model performance with 95% confidence intervals when using input images of sizes of 128×128, 256×256, 512×512, and 1024×1024. For COVID-GMIC, we started with a size of 256×256 since an image with resolution of 128×128 pixels results in saliency maps that are too small to generate meaningful ROI patches. We report AUCs for predicting the risk of deterioration within 24, 48, 72, and 96 hours. When evaluating the deterioration risk curves, we report the concordance index with a reference time of 96 hours, as well as the average of the index over all possible reference times (3, 12, 24, 48, 72, 96, 144, and 192 hours).

		AUC / PR AUC				Concordance index	
		24 hours	48 hours	72 hours	96 hours	96 hours	Average
DenseNet-121	128×128	0.663 (0.602, 0.733) / 0.214 (0.119, 0.284)	0.688 (0.633, 0.749) / 0.300 (0.198, 0.376)	0.700 (0.649, 0.753) / 0.370 (0.279, 0.448)	0.728 (0.685, 0.781) / 0.453 (0.364, 0.533)	0.700 (0.667, 0.734)	0.700 (0.672, 0.736)
	256×256	**0.698** (0.632, 0.763) / **0.218** (0.153, 0.310)	**0.721** (0.668, 0.778) / 0.310 (0.207, 0.382)	**0.719** (0.670, 0.773) / **0.390** (0.318, 0.486)	**0.748** (0.701, 0.795) / **0.469** (0.392, 0.562)	0.701 (0.666, 0.738)	0.698 (0.663, 0.734)
	512×512	0.682 (0.617, 0.749) / 0.208 (0.111, 0.267)	0.710 (0.658, 0.764) / **0.318** (0.238, 0.422)	0.709 (0.656, 0.764) / 0.383 (0.286, 0.459)	0.732 (0.686, 0.780) / 0.441 (0.353, 0.516)	**0.705** (0.673,0.739)	**0.701** (0.669,0.735)
	1024×1024	0.680 (0.619, 0.742) / 0.180 (0.101, 0.230)	0.709 (0.657, 0.763) / 0.278 (0.185, 0.344)	0.716 (0.666, 0.766) / 0.369 (0.269, 0.442)	0.739 (0.694, 0.787) / 0.441 (0.353, 0.516)	0.701 (0.668, 0.734)	0.696 (0.664, 0.729)
COVID-GMIC	256×256	0.664 (0.593, 0.734) / 0.202 (0.101, 0.260)	0.688 (0.630, 0.747) / 0.263 (0.172, 0.326)	0.699 (0.651, 0.750) / 0.342 (0.253, 0.414)	0.728 (0.684, 0.774) / 0.424 (0.343, 0.492)	0.712 (0.679, 0.744)	0.707 (0.675, 0.741)
	512×512	**0.700** (0.635, 0.765) / **0.210** (0.154, 0.298)	0.714 (0.661, 0.769) / 0.300 (0.205, 0.370)	0.714 (0.671, 0.766) / **0.389** (0.314, 0.481)	0.733 (0.690, 0.780) / 0.443 (0.354, 0.515)	**0.713** (0.679,0.748)	**0.708** (0.675, 0.742)
	1024×1024	0.695 (0.630, 0.763) / 0.200 (0.121, 0.258)	**0.716** (0.661, 0.767) / **0.302** (0.230, 0.394)	**0.717** (0.663, 0.764) / 0.374 (0.289, 0.447)	**0.738** (0.692, 0.780) / **0.439** (0.368, 0.522)	0.686 (0.650, 0.720)	0.685 (0.648, 0.717)

Impact of different transfer learning strategies. In data-scarce applications, it is crucial to pretrain deep neural networks on a related task for which a large dataset is available, prior to fine-tuning on the task of interest [68, 69]. Given the relatively small number of COVID-19 positive cases in our dataset, we investigate the impact of different weight initialization strategies on our results. Specifically, we compare three strategies: 1) initialization by He et al. [70], 2) initialization with weights from models trained on natural images (ImageNet [66]), and 3) initialization with weights

from models trained on chest X-ray images (ChestX-ray14 dataset [59]). We apply the initialization procedure to all layers except the last fully connected layer, which is always initialized randomly. We then fine-tune the entire network on our COVID-19 task.

Based on results shown in Supplementary Table 5, fine-tuning the network from weights pretrained on the ChestX-ray14 dataset is the most effective strategy for COVID-GMIC. This dataset contains over 100,000 chest X-ray images from more than 30,000 patients, including many with advanced lung disease. The images are paired with labels representing fourteen common thoracic observations: atelectasis, cardiomegaly, effusion, infiltration, mass, nodule, pneumonia, pneumothorax, consolidation, edema, emphysema, fibrosis, pleural thickening, and hernia. By pretraining a model to detect these conditions, we hypothesize that the model learns a representation that is useful for our downstream task of COVID-19 prognosis.

Supplementary Table 5: Model performance with 95% confidence intervals across three different initialization strategies: random initialization, initialization with the weights of the model pretrained on ImageNet [66] and initialization with the weights of the model pretrained model on the ChestX-ray14 dataset [59]. We report AUCs for each time window in the outcome classification task. When evaluating the deterioration risk curves, we report the concordance index with a reference time of 96 hours, as well as the average of the index over all discretized times (3, 12, 24, 48, 72, 96, 144, and 192 hours).

		AUC / PR AUC				Concordance index	
		24 hours	48 hours	72 hours	96 hours	96 hours	Average
DenseNet-121	Random	0.687 (0.625, 0.753) / 0.178 (0.105, 0.222)	0.699 (0.648, 0.754) / 0.258 (0.177, 0.315)	0.693 (0.642, 0.747) / 0.326 (0.236, 0.388)	0.705 (0.660, 0.752) / 0.386 (0.298, 0.449)	0.649 (0.614, 0.686)	0.648 (0.613, 0.685)
	ImageNet	**0.701** (0.639, 0.761) / 0.206 (0.117, 0.260)	**0.722** (0.668, 0.776) / 0.299 (0.197, 0.366)	**0.719** (0.670, 0.772) / 0.365 (0.264, 0.436)	**0.745** (0.701, 0.789) / 0.444 (0.349, 0.513)	0.686 (0.652, 0.720)	0.683 (0.651, 0.715)
	ChestX-ray14	0.687 (0.616, 0.755) / **0.216** (0.155, 0.317)	0.709 (0.651, 0.765) / **0.315** (0.239, 0.419)	0.710 (0.657, 0.760) / **0.373** (0.300, 0.464)	0.736 (0.690, 0.781) / **0.454** (0.384, 0.542)	**0.705** (0.673,0.739)	**0.701** (0.669,0.735)
COVID-GMIC	Random	0.675 (0.609, 0.743) / 0.174 (0.101, 0.223)	0.671 (0.614, 0.725) / 0.227 (0.146, 0.277)	0.686 (0.640, 0.732) / 0.290 (0.214, 0.345)	0.708 (0.668, 0.752) / 0.352 (0.276, 0.410)	0.643 (0.606, 0.678)	0.640 (0.604, 0.673)
	ImageNet	0.694 (0.635, 0.757) / 0.195 (0.110, 0.252)	0.709 (0.657, 0.761) / 0.258 (0.165, 0.319)	extbf0.724 (0.673, 0.769) / 0.347 (0.263, 0.416)	0.737 (0.696, 0.782) / 0.433 (0.354, 0.506)	0.684 (0.652, 0.717)	0.680 (0.649, 0.711)
	ChestX-ray14	**0.695** (0.626, 0.757) / **0.200** (0.142, 0.283)	**0.716** (0.659, 0.768) / **0.302** (0.228, 0.400)	0.717 (0.672, 0.769) / **0.374** (0.302, 0.463)	**0.738** (0.690, 0.783) / **0.439** (0.368, 0.532)	**0.713** (0.679,0.748)	**0.708** (0.675,0.742)

Supplementary Note 4: Model selection

We describe our model selection procedure used throughout the paper in Algorithm 1. For the ablation study in Supplementary Table 3, we control the size of the dataset by setting the parameter u to 12.5, 25 and 50. Specifically, in that experiment, we randomly sampled $u\%$ of the training set $\mathcal{D}_t$ as the "universe" $\mathcal{U}$ that our model used for training and validation.

Algorithm 1 Model selection

Input: training set $\mathcal{D}_t$, test set $\mathcal{D}_s$, universe fraction $u \in [0, 100]$, and a predictive model $\mathcal{M}$
Output: a^* performance of $\mathcal{M}$ evaluated on $\mathcal{D}_s$

```
𝒰 = randomly sample u% of data from 𝒟_t
Φ = 30 randomly sampled configuration of hyperparameters of the ℳ
for each hyperparameter configuration φ_i ∈ Φ do
    for j ∈ {1, 2, 3} do
        draw a random seed r_j
        𝒰_t^j, 𝒰_v^j = universe 𝒰 split into training and validation subset using the random seed r_j
        ℳ_ij = trained ℳ using hyperparameter configuration φ_i on 𝒰_t^j
        a_ij = performance of ℳ_ij evaluated on 𝒰_v^j
    end for
    a_i = 1/3 Σ_{j=1}^{3} a_ij
end for
𝒜 = {a_i | ∀φ_i ∈ Φ}
ℬ = {ℳ_ij | ∀a_i ∈ top-3(𝒜)}
ℳ* = an equally weighted ensemble of all models in ℬ
a* = performance of ℳ* on 𝒟_s
return a*
```

Supplementary Note 5: ROI retrieval algorithm

We describe the algorithm used to retrieve the ROI patches in Algorithm 2. In all experiments, we set $H = W = 1024$, $h = w = 32$, $h_c = w_c = 256$, $\mathbb{T}_a = \{24, 48, 72, 96\}$, and $K = 6$.

Algorithm 2 ROI retrival

Input: chest X-ray image $\mathbf{x} \in \mathbb{R}^{H,W}$, saliency maps $\mathbf{A} \in \mathbb{R}^{h,w,|\mathbb{T}_a|}$, number of ROI patches K
Output: a set of retrieved ROI patches $O = \{\tilde{\mathbf{x}}_k | \tilde{\mathbf{x}}_k \in \mathbb{R}^{h_c,w_c}\}$

```
O = ∅
for each time window t ∈ 𝕋_a do
    Ã^t = min-max-normalization(A^t)
end for
A* = Σ_{t∈𝕋_a} Ã^t
l denotes an arbitrary h_c (h/H) × w_c (w/W) rectangular patch on A*
criterion(l, A*) = Σ_{(i,j)∈l} A*[i, j]
for each 1, 2, ..., K do
    l* = argmax_l criterion(l, A*)
    L = position of l* in x
    O = O ∪ {L}
    ∀(i, j) ∈ l*, set A*[i, j] = 0
end for
return O
```
